# Studying the Relationship of Intermittent Fasting and β-Amyloid in Animal Model of Alzheimer’s Disease: A Scoping Review

**DOI:** 10.3390/nu12103215

**Published:** 2020-10-21

**Authors:** Muhammad Luqman Nasaruddin, Syarifah Aisyah Syed Abd Halim, Mohd Amir Kamaruzzaman

**Affiliations:** 1 Department of Biochemistry, Faculty of Medicine, Universiti Kebangsaan Malaysia Medical Centre, Level 17, Preclinical Building, Jalan Yaacob Latif, Bandar Tun Razak, Kuala Lumpur 56000, Malaysia; 2 Department of Anatomy, Faculty of Medicine, Universiti Kebangsaan Malaysia Medical Centre, Level 18, Preclinical Building, Jalan Yaacob Latif, Bandar Tun Razak, Kuala Lumpur, 56000 Malaysia; syarifahaisyah@ukm.edu.my (S.A.S.A.H.); mohdamir@ukm.edu.my (M.A.K.)

**Keywords:** intermittent fasting, alternate day fasting, time-restricted feeding, Alzheimer’s disease, β-amyloid

## Abstract

We examined the evidence for intermittent fasting (IF) as a preventative tool to influence β-amyloid in animal models of Alzheimer’s disease (AD). A Scopus, Ovid, PubMed, and Web of Science (WoS), search yielded 29 results using the keywords “amyloid beta”, “intermittent fasting”, “intermittent caloric restriction”, “alternate day fasting”, “modified alternate-day fasting”, “time-restricted feeding”, “Ramadan fast”, “intermittent calori* restriction”, “intermittent restrictive diet”, and “Alzheimer*”. Five research articles addressed directly the effects of intermittent fasting on β-amyloid levels in animal models of AD: alternate day fasting (ADF) and time-restricted feeding (TRF) methods were incorporated in these studies. The study designs were found to be heterogeneous. Variations in the levels of β-amyloid peptides or plaque in either the hippocampus, cortical areas, or both in animals following dietary intervention were observed as compared to the ad libitum group. Non-significant changes were observed in three studies, while two studies interestingly demonstrated amelioration and reduction in β-amyloid levels. Given the conflicting results obtained from this study, significant care has to be taken into consideration before the protocol can be applied as a preventative approach to treat Alzheimer’s disease. Longitudinal research is warranted to fully grasp how dietary habits can help alleviate the disease either through upstream or downstream of AD pathology.

## 1. Introduction

Intermittent fasting has recently gained popularity owing to several health benefits associated with its practice [1]. It is an alternative means used to achieve weight loss as opposed to the traditional method of undergoing caloric restriction (20–40% reduction in daily caloric intake) and/or physical activity. This lifestyle intervention is not a type of diet in a conventional sense, but a pattern that repetitively cycles between periods of fasting and intervened with unrestricted eating periods. Fasting in contrast to starvation is the voluntary abstinence from food, while the latter is neither voluntary nor controlled and is a state of chronic nutritional insufficiency.

There are several different variants of intermittent fasting: every other day/alternate day fasting (ADF) (which consists of fasting for a full 24 h period and an unrestrained food intake the following day), periodic fasting (fasting periods of two days to three weeks in duration) [2], time-restricted feeding (TRF) (an eating pattern in which food intake is restricted to a time window of 8 h or less) [3], and intermittent energy restriction (alternating periods of restricted and unrestricted energy consumption) [4]. Studies in human subjects have demonstrated an improvement in health indicators both in healthy and diseased conditions. These health outcomes are not limited to solely weight control but in a plethora of other circumstances including cardiovascular disorders, metabolic syndrome, and cancer [2,5,6,7,8,9,10,11,12].

Recent studies have indicated that intermittent fasting showed promising protective effects against neurodegeneration. Despite significant amelioration observed in animal models of Parkinson’s disease (PD), Huntington’s disease (HD), and traumatic brain injury (TBI) following dietary intermittent protocols [13,14,15,16], little is known of the outcome in AD, more specifically on β-amyloid in AD brain. AD is characterized by progressive cognitive dysfunction and is diagnosed based on the definitive hallmark presence of β-amyloid plaque and neurofibrillary tangles in the brain [17,18]. It is thought to be caused by the overproduction and/or failure of β-amyloid clearance, which led to its accumulation [19,20]. β-amyloid is produced by neurons and astrocytes as well as non-neural tissues [21]. The production of amyloidogenic β-amyloid peptides requires sequential cleavage of the amyloid precursor protein (APP) by β-secretase at the N-terminus and γ-secretase at the C-terminus of the β-amyloid that produces monomers (aβ-40 and β-42 are the most abundant forms) [22,23]. β-amyloid monomers then rapidly aggregate into fibril forms that deposit into the amyloid plaques. The genetic heritability of AD has shown that the mutation in one of the genes that encode for APP and presenilin 1 and 2 have resulted in β-amyloid overproduction [24,25]. The “amyloid cascade hypothesis”, which has directed studies in dementia for decades, postulates that the fundamental cause of AD is the deposition of extracellular β-amyloid peptides [26]. However, this hypothesis has been met with resistance and controversy owing to several findings that indicated otherwise [27,28,29].

Despite these discoveries, the study of β-amyloid is still relevant. Recent machine learning algorithms have demonstrated the importance of elevated β-amyloid and tau biomarkers as good predictors for early dementia status [30]. Moreover, the ratio of aβ-42/40 was found to drive tau pathology in a neural cell culture model of AD [31]. Furthermore, the oligomeric form of β-amyloid has been found to induce early and widespread proteomic alterations in induced pluripotent stem cells-derived neurons within 24 h of its uptake [32]. This provides a repository of new biomarkers and strategies that may be key in mediating the onset and progression of AD.

To date, there is no cure to halt or prevent AD. As AD is influenced by several lifestyle factors in addition to genetic factors, it is only fitting that lifestyle interventions could provide assistance in improving or maintaining cognitive functions and reduce neuronal pathological burdens. To date, intermittent fasting has not been explored as a potential therapy in human AD subjects. However, we do have evidence to believe that fasting may be a potent practical tool against the disease given its ability to improve symptoms of AD, as seen in a number of studies involving ketone body namely, β-hydroxybutyrate [33,34,35,36,37].

This scoping review was undertaken to summarize published data on the relationship between intermittent fasting and β-amyloid in animal models of AD. This scoping review is conducted to better understand how the latter could be affected by dietary intervention whilst pointing out the disparities of findings in the literature. The summary shall be provided using a systematic search across many different databases. We hypothesize that intermittent fasting can influence β-amyloid deposition in AD brains.

## 2. Materials and Methods

A systematic search was undertaken in this scoping review to identify and map out relevant and pertinent articles related to the current study. Peer-reviewed and full-text English articles were gathered from a time frame as early as 1960 to May 2020 in electronic databases including: Scopus, Ovid, PubMed, and Web of Science (WOS). The search terms used together with the Boolean operators AND and OR were as follows: “amyloid beta” OR “beta-amyloid” OR “β-amyloid” OR “amyloid-β” AND “intermittent fasting” OR “intermittent caloric restriction” OR “alternate day fasting” OR “modified alternate-day fasting” OR “time-restricted feeding” OR “Ramadan fast” OR “intermittent restrictive diet” AND “Alzheimer*”. The inclusion criteria of this study were that (1) only primary articles were considered, (2) only experimental animal studies of AD were shortlisted, (3) the intervention applied to these animals was only those that underwent intermittent fasting, and (4) the effects of β-amyloid plaque or peptides were measured. In contrast, the exclusion criteria for the study were (1) studies that involved humans, (2) not specific to AD, (3) the intervention applied to the study was continuous calorie/energy restriction or starvation, and (4) the effects of β-amyloid plaque or peptides were not measured. All titles and abstracts from the search results were initially screened and independently critiqued by all authors. Following the inclusion and exclusion criteria, the articles were shortlisted for eligibility upon reaching a consensus by all authors.

## 3. Results

The literature search identified a total of 29 articles from these databases. Sixteen articles were found to be duplicates (based on the title and abstract) and were subsequently removed. The 13 remaining articles were then screened and analyzed with much depth for eligibility. Five articles met the inclusion criteria and were included in the current review. A summary of the data extracted from the five articles can be found in Table 1.

Several different transgenic mice models were used in the selected review. One study incorporated 5xFAD mice that carry five familial AD mutations: Swedish (K670N), M671L), Florida (1716V), and London (V7171) mutations in human amyloid precursor protein (APP695) and two mutations (M146L and L286V) in the human presenilin 1 protein [38]. One study employed a triple transgenic AD mice model with one harboring Swedish double mutation, beta-amyloid precursor protein (betaAPPSwe), presenilin-1 (PS1M146V), and human tauP301L [42], while the other was an APP knock-in mice (APP^NL-G-F^) that developed several features of AD [39]. Furthermore, one other study employed a double transgenic APP-PS1 mouse [41]. All of these mice were designed to increase the deposition rate of β-amyloid in the brain with some as early as two months old [38]. Interestingly, only one study was found to employ ovariectomized female rats instead, and they were infused with β-amyloid peptides into their hippocampus [40]. The age of the mice and rats at the start of the intervention varied from study to study. Three studies started the intervention as early as two to three months old [38,40,42], while the other at five months old [41], and lastly at 12 months old [39].

Three studies underwent the ADF method where food was deprived for a period of 24 h [38,41,42], while the remaining two studies employed a TRF protocol of 3 h per day [40] and 2 days per week of food deprivation [39]. The intervention duration was also found to be wide-ranging across studies. For the ADF protocol, the shortest duration was 4 months [38], followed by 5 months [41], and the longest at 14 months [42]. The time-restricted protocol of 3 h per day started at the beginning of the dark cycle (7 to 10 PM), which corresponds to the morning for humans for a period of 2 months [40], while the 2 days per week food restriction was conducted for 9 months [39].

The majority of the studies reported in this review utilized immunohistochemical (IHC) analyses for the detection of β-amyloid plaque formation [38,39,40,41]. However, they differed in terms of the staining or primary antibodies used to detect β-amyloid. At least one used thioflavin-s staining [38], while others used monoclonal or polyclonal aβ-42 primary antibodies [39,41]. Moreover, one study did not specify in detail the types of primary antibody used for IHC [40]. Additionally, two studies employed ELISA with either aβ-42 alone or with aβ-40 [38,42].

The effect of intermittent fasting on the changes of β-amyloid deposition in the hippocampus and/or other cortical areas of the brain was found to be conflicted. β-amyloid levels in the hippocampus of the triple transgenic (3xTgAD) AD mice undergoing ADF were found to be not significantly different (Aβ-42 and Aβ-40) from the levels found in littermates that were fed ad libitum [42]. The observation was also true as demonstrated in 5xFAD mice that underwent a similar protocol [38]. Thioflavin-S positive plaque levels in both 5xFAD fed ad libitum and 5xFAD-ADF were found to be comparable and non-significantly different [38]. Subsequent measurement of the individual β-amyloid peptides via ELISA in both of these brain areas demonstrated no significant changes as well [38]. Interestingly, one TRF study on a knock-in mouse model (APP^NL-G-F^) saw a non-significant trend towards lower amounts of β-amyloid accumulation in APP^NL-G-F^ mice on the protocol as compared to APP^NL-G-F^ fed ad libitum [39]. In contrast, only in a double transgenic mouse model of AD (APP/PS1) were significant differences of β-amyloid levels observed in the cortical areas between mice fed ad libitum and ADF [41]. The same result was also demonstrated in non-transgenic ovariectomized rats that underwent TRF [40]. These rats exhibited less β-amyloid deposition in the hippocampus as compared to those that were fed ad libitum [40].

Moreover, a number of different cognitive and/or behavioral tests were also employed in these studies to gauge the effects of intermittent fasting on AD mouse/rat models. The Morris water maze test was employed with most if not all of the animal models that underwent intermittent fasting to demonstrate improved goal latency [39,40,41,42], as well as in the probe trial scores of the swimming task [40,41,42], in comparison to ad libitum fed littermates. Furthermore, the knock-in AD mouse model [39] that underwent ADF showed increased performance in the Y-maze test as compared to its counterparts that were fed ad libitum. Like the Morris water maze test, the knock-in AD mice with intermittent fasting had lower goal latency scores in their 2-day water maze test as opposed to the non-fasted ad libitum littermates [39]. Additionally, ovariectomized AD rats that underwent TRF showed reduced latency period in the passive avoidance test in contrast to those that were fed ad libitum [40]. In contrast, 5xFAD mice that underwent ADF demonstrated increased anxiety-like behavior following the light/dark box test and deteriorating short term memory in the novel object location (NOL) experiment as compared to their non-fasted, ad libitum fed 5xFAD mice [38]. Additionally, while the fasted 5xFAD mice demonstrated no signs of mobility deficits in the open field test [38], aged triple transgenic mice-AD mice with ADF had improved distance traveled and ambulatory count scores as compared to transgenic mice that were fed ad libitum [42].

## 4. Discussion

This scoping review suggests that evidence of restriction in the frequency of feeding and reduction in β-amyloid levels is still in its infancy. Unlike studies that employed continuous calorie restriction in a transgenic mouse model of AD where the results demonstrated a significant reduction in β-amyloid burden [43,44,45,46], only two studies reviewed here [40,41] advocated a positive relationship between intermittent fasting and reduction in plaque deposition in the brain. The remaining three studies showed no significant changes or reductions in β-amyloid plaque [38,39,42].

This led us to hypothesize that genetic background and/or transgene expressions in mice played a strong influence on the final molecular outcome following the intervention. A recent study has shown that plaque burdens and distribution among transgenic lines are highly heterogeneous, and changes across their lifespan are line- and region-dependent [47]. The study showed that the dense core plaque burdens were varied by more than an order of magnitude between the lines [47]. The study further reported that the median cortical plaque burden in 15-month-old 5xFAD mice was 4.5 times that of 21-month old Tg2576 and 15-month old rTg9191 mice [47]. Taking this into consideration, we feel that the intervention fails to produce positive results in these mice owing to the burden that may be too overwhelming as compared to those studies that employed only a double transgenic mouse model and rats that were directly infused with β-amyloid peptides. This goes to show that the intervention may have a much higher success rate in sporadic AD cases in humans as humans have far fewer plaque build-ups and smaller size as compared to familial, animal models of AD [47,48,49].

In addition, one startling finding that we further noted was that employing intermittent fasting feeding at an early or prodromal stage in mice with multiple transgenes like 5xFAD has resulted in an exacerbated neuroinflammatory activity in the cortex of these mice [38]. A 2-fold increase in Iba1-positive microglial cells coupled with elevated levels of tumor necrosis factor-alpha (TNF-α), a potent pro-inflammatory cytokine, were detected in the 5xFAD in comparison to controls fed ad libitum [38]. This observation was further accompanied by a trend in increased anxiety-like behaviors [38]. This negative effect was also demonstrated in a poly(inosinic:cytidylic)(I:C) model of inflammation [50]. ADF was found to amplify the levels of circulating cytokines and aggravated sickness behavior in response to Poly(I:C) [50]. Moreover, the same dietary protocol failed to demonstrate protective effects against colitis and related behavioral disorders in contrast to TRF and intermittent energy restriction (IER) [51]. This could mean that different dietary protocols may have a range of different effects, with some potentially aggravating pre-existing pathology such as the case of ADF as applied to 5xFAD mice.

Furthermore, it is however surprising to note that while the outcome for changes in β-amyloid levels as a result of the intervention is mixed in general, cognitive dysfunction was found to be ameliorated in these animals. Except for one study involving 5xFAD mice [38], intermittent fasting protected these animals by improving defunct spatial learning and memory as compared to those that were fed ad libitum [39,40,41,42]. Transgenic and knock-in mice, as well as the β-amyloid-infused AD-rats that underwent intermittent fasting, showed cognitive improvements in the Y-maze test, a 2-day water maze test, passive avoidance test, and Morris water maze test in comparison to either wild-type or transgenic/knock-in/infused-AD animals fed ad libitum [39,40,41,42]. The current understanding of the correlation between plaque load and cognitive impairment in humans, on the whole, has been rather conflicted. Recent findings have shown that plaque loads do not correlate well with cognitive impairment in humans with AD [52,53]. Further studies have demonstrated extensive β-amyloid pathology in cognitively normal aged individuals [54,55]. Fresh evidence has suggested that cognitive outcomes are much closely related to tau proteins than they are to β-amyloid plaques [56]. This provides a plausible explanation as to how some humans can tolerate a high β-amyloid load without cognitive impairment [42]. Though this is the case, we fail to see a reduction in the levels of tau proteins in the 3xtgAD mice model undergoing ADF as compared to controls fed ad libitum that could explain the improved cognitive dysfunctions in this group [42]. A similar observation can be said for in the β-amyloid-infused AD rats that underwent intermittent fasting [40]. This begs the question of whether or not intermittent fasting is protecting these animals via a different and unelucidated mechanistic pathway foreign from the actions or presence of tau and/or β-amyloid?

To date, no study has explored the potentiality of intermittent fasting in human AD subjects. However, its application in one relatable study of aged individuals has resulted in positive outcomes. Elderly people with mild cognitive impairment (MCI) who regularly practice intermittent fasting showed a significant increase in cognitive scores in a wide range of psychological domains in contrast to irregular and nonregular fasters [57]. These people showed a significant increase in the antioxidant superoxide dismutase levels with significantly lower DNA damage events [57]. They were also found to have reduced malondialdehyde and c-reactive protein levels as compared to levels at baseline [57]. These subjects interestingly presented with a reversion towards healthy cognitive states upon 36 months period follow up [57]. This provides preliminary evidence for potential neuroprotective and neurocognitive effects of intermittent fasting in people with AD.

Intermittent fasting confers neuroprotective effects through increased neurotrophic factor signaling and activation of autophagy [58,59,60,61]. The current review suggests that intermittent fasting restored Aquaporin-4 (AQP4) levels which is crucial for AQP4-dependent glial and lymphatic pathway systems that are key in the removal of soluble β-amyloid from the interstitium [41]. Moreover, the study by Liu and colleagues [39] reported hippocampal neuronal network adaptations mediated by SIRT-3 in APP^NL-G-F^ knock-in mice that underwent intermittent fasting that is associated with reduced anxiety-like behaviors and elevated hippocampal-dependent memory, independent of β-amyloid plaque deposition. These taken together provide plausible evidence, though limited, that intermittent fasting may preserve synaptic functions even in the presence of β-amyloid.

Our finding has to be taken with great caution. The limitation of this review was that it only covered five studies related to intermittent fasting and β-amyloid deposition. The study design in these studies was found to be heterogeneous in nature whereby the dietary protocols, types of experimental animal used, age at onset, and the duration of the study are wide-ranging. This makes it difficult for us to interpret with confidence and definitively on the effectiveness of intermittent fasting as a treatment/preventative tool to influence or reduce β-amyloid levels in AD.

Bear in mind again that the animal models reported here are mostly transgenic in nature. These animals possess a combination of multicopy transgenes, amyloid precursor protein (APP), and mutations of Presenilin 1/2 (PS1/2) and/or tau, that seek to express high levels of β-amyloid. The 5xFAD model, for example, is a complex model created from combining three APP and two PS1 mutations that cause rapid onset of β-amyloid plaque accumulation as early as two months of age. Similarly, the knock-in mouse model as reported here, while devoid of APP overexpression like 5xFAD, produces progressive plaque development starting at 2 months old and becomes saturated at 7 months of age. In contrast, occasional β-amyloid can be seen as early as 6 months old for double and triple mice models and progresses further with age. These taken together make it difficult to collectively and conclusively confirm the success or failure of the treatment or intervention if it is only measured at one single time point (i.e., at the end of the intervention period). Intermittent fasting, in this case, may have been targeting varying pathways depending on when we measured the β-amyloid as well as the genetic background of the mice we are testing against. This could partially explain why the significance of the findings reported in this review was not consistent.

A longitudinal approach looking into the levels of β-amyloid at multiple time points would help in overcoming this problem. By studying the temporal/or age-related changes occurring at the molecular level, as well as its corresponding behavioral and/or cognitive changes at 2, 4, or 6 months of the fasting period, for example, would assist greatly in gauging how well intermittent fasting is exerting its effect on AD pathology. Furthermore, applying the metabolomics platform during these time points would provide invaluable information on crucial biomarkers that may be key in elucidating the neuroprotective mechanisms of intermittent fasting. Additionally, this study should comprise of several different forms of interventions (ADF or TRF) tested in tandem to observe the best protocol and circumstances that could make it suitable without posing a threat to the wellbeing of the animal or exacerbating pre-existing pathology. Moreover, fixing the duration of the fasting periods would be highly ideal in this case given the potential usage of this intervention in multiple different models.

Furthermore, as the different studies reported here provided different methods of analyzing the levels of β-amyloid (i.e., IHC or ELISA), this makes it slightly problematic to properly compare the data. Future studies should present their IHC findings using percentages of the area that occupied β-amyloid reactivity rather than using the total number or the average number of plaques present. Quantification methods of these immunoreactivity readings have to be standardized using a single machine or software to ensure intra/inter-laboratory reproducibility. This also extends to how the sections were collected and prepared. Moreover, for ELISA, data presentation using a fixed scale is rather crucial. These are needed to allow for consistency of future study designs as well as to allow direct comparison across different studies.

Additionally, the effect of intermittent fasting on neurofibrillary tangle expression should also be further explored. Other than the works reported in the current review, there is no other study on the effects of intermittent fasting on tau pathology. To date, there is only one study that measured the effect of the ketone body, β-hydroxybutyrate (a known by-product of fasting), on tau tangles. The study demonstrated a reduction in high-fat diet (HFD)-induced aggregates of tau tangles in apolipoproteinE4 (APOE4)-deficient mice following its administration [62]. This provides promising proof that the application of intermittent fasting is highly relevant when using an appropriate model.

Moreover, as these mice mimic the rare genetic form that develops either plaque or in a combination with neurofibrillary tau tangles, the use of the sporadic AD model is highly warranted for future studies. One recent model that was made possible by intra-cerebroventricular or intraperitoneal injection of streptozotocin (STZ), a glucosamine-nitrosourea, in the brain of rodents has been proposed as a representative model of sporadic AD [63]. The administration of STZ has resulted in an insulin-resistant brain state that shares common features of sporadic AD in humans. This includes spatial learning deficit, memory impairment, increased cerebral aggregated β-amyloid fragments and β-amyloid deposits, total tau proteins, neuroinflammation, oxidative stress, cholinergic deficits, and glucose hypometabolism [63]. To date, the application of intermittent fasting on this model was only directed towards modulation of the diabetic syndrome, and not neurodegeneration [64]. This provides a fresh avenue to study intermittent fasting in the context of Alzheimer’s disease in a much relevant setting.

Additionally, studying the effects of intermittent fasting on neuroinflammation could also increase our understanding of how dietary intervention can influence AD pathology. While the direct study of intermittent fasting, AD, and neuroinflammation is lacking, there are previous relatable studies that could provide preliminary evidence that can be considered for future undertakings. One such study has reported that intermittent fasting pretreatment provided sustained neuroprotection against inflammation and lowers cognitive impairments in a rat model of vascular dementia [65]. Additionally, another study has demonstrated the amelioration of cognitive deficits in a rat model of sepsis [66]. The areas of future research could be extended by looking into the inflammatory effects caused by activated microglia owing to the presence of β-amyloid and/or tau and how intermittent fasting can improve the condition in a sporadic model of AD.

Moreover, considering the high prevalence of women with AD, it is best to invest in future studies on the effect of sex differences in AD and its relation to intermittent fasting. There is only one study reported that directly compared the differences. However, the comparison was limited to only cognitive changes following intermittent fasting, and not β-amyloid and/or tau pathology. The study exhibited poor or longer escape latency and path lengths traversed in the Morris water maze test for female 3xtg-AD ad libitum fed mice in comparison to male 3xtg-AD [42]. Additionally, both male and female 3xtg-AD mice demonstrated improved performance in the hidden platform test following intermittent fasting [42]. The application of intermittent fasting on sex differences is pertinent to allow for targeted intervention to be introduced prior to the clinical manifestations of AD in both males and females.

Furthermore, studies on the effect of dietary protocols on β-amyloid protein clearance and degradation pathway have to be lengthened since sporadic AD is believed to arise due to the failure of β-amyloid clearance [67]. Besides, this work can further be extended by looking into the outcome of intermittent fasting on the gut microbiome and how it may synergistically improve neurodegeneration. A recent study has demonstrated that intermittent fasting ameliorated diabetes-induced cognitive impairment and is mediated by gut microbiota [68]. Lastly, studies on the practicability of intermittent fasting versus caloric restriction in the current context are still limited. This warrants for further investigation to be conducted in animals and humans looking at the differentially activated pathways from these two lifestyle interventions.

## 5. Conclusions

This scoping review summarizes the current knowledge and understanding of intermittent fasting and its relation with β-amyloid deposition in experimental animal models of AD. Findings gained from this study proved to be difficult to be generalized given the limited number of studies reviewed and the heterogeneity of the study designs. However, there appears to be a relationship between intermittent fasting and β-amyloid deposition which warrants additional work in the area and provides a window of opportunity for how the intervention could potentially work in humans. A future longitudinal study looking into several different intermittent fasting protocols in tandem, on a specific animal model coupled with metabolomics analyses, could provide novel mechanistic insights as to how the two are connected. Further work on tau pathology and neuroinflammation have to be considered. The success of future studies could demonstrate intermittent fasting as a cheap, viable, and potent preventative tool against AD.

## Figures and Tables

**Table 1:** The summary of data extracted from eligible articles.

Year	Author	Animal Model	Sex	Age of Animal at the Start of Intervention	Intervention Duration	Mode of Intermittent Fasting (Regime)	Method of Analysis	Area of the Brain Measured	Results	Behavioral/Cognitive Changes
2020	[38]	5xFAD (Transgenic mouse model)	(F)	2 months	4 months	(ADF) (Treatment)	(IHC) (ELISA)	Cortex and Hippocampus	No significant changes in β-amyloid levels between intermittent fasting (IF) vs. ad libitum group	1. 5xFAD-ADF displayed increased levels of anxiety than non-transgenic mice fed ad libitum in the light/dark box test. 2. 5xFAD-ADF experienced a significant decline in short term memory than non-transgenic mice fed ad libitum in the Novel Object Recognition and Location (NOR & NOL) tests. 3. 5xFAD-ADF displayed a non-significant decrease in short term memory than 5xFAD-ad libitum in the Novel Object Location (NOL) test. 4. 5xFAD-ADF presented no mobility deficits in the open field test.
2020	[39]	3xAD (Knock-in mouse mode) (APP^NL-G-F^))	(M)	12 months	9 months	(TRF; 2 days/week) (Treatment)	IHC	Hippocampus	A non-significant trend towards lower amounts of β-amyloid levels between IF vs. ad libitum group	1. APP^NL-G-F^-TRF performed significantly better than APP^NL-G-F^-ad libitum in the Y-Maze test. 2. Mice that are being maintained on the TRF diet (both WT and APP^NL-G-F^) demonstrated lower goal latency time in the 2-day water maze test.
2018	[40]	Ovariectomized Sprague Dawley rats (Direct infusion with aβ-42, aβ-45, aβ25-35)	(F)	2 months and 3 weeks	2 months	TRF (3 h/day) (Prophylactic)	IHC	Hippocampus	Significant reduction in β-amyloid levels between IF vs. ad libitum group	1. Ovariectomized-AD-TRF ameliorated short-term memory deficit in the passive avoidance test. 2. Ovariectomized-AD-TRF showed improved spatial memory than Ovariectomized-AD-ad libitum in the Morris Water Maze test.
2017	[41]	APP/PS1 (Transgenic mouse model)	(M&F)	5 months	5 months	ADF (Treatment)	IHC	Cerebral Cortex	Significant reduction in β-amyloid levels between IF vs. ad libitum group	1. APP-PS1-ADF demonstrated improved spatial memory than APP/PS1-ad libitum in the Morris Water Maze test.
2007	[42]	3xtgAD (Transgenic mouse model)	(M&F)	3 months	14 months	ADF (Treatment)	ELISA	Hippocampus	No significant changes in β-amyloid levels between IF vs. ad libitum group	1. 3xAD-ADF displayed an improved age-related decrease in ambulatory counts and distance traveled in the open field test. 2. 3xAD-ADF displayed improved performance in comparison to 3xAD-ad libitum in the Morris Water Maze test.

Abbreviation: M (Male), F (Female), IHC (immunohistochemical analysis), ELISA (enzyme-linked immunosorbent assay), IF (intermittent fasting), ADF (alternate day fasting), TRF (time restricted feeding), WT (Wild type), 5xFAD-ADF (5xFAD transgenic mice underwent alternate day fasting), 5xFAD-ad libitum (5xFAD transgenic mice fed ad libitum), APP^NL-G-F^ (knock-in mouse model ), APP^NL-G-F^-TRF (knock-in mice underwent time restricted feeding), APP^NL-G-F^-ad libitum (knock-in mice fed ad libitum), Ovariectomized-AD-TRF (non-transgenic ovariectomized rats infused with β-amyloid, underwent time restricted feeding), Ovariectomized-AD-ad libitum (non-transgenic ovariectomized rats infused with β-amyloid, fed ad libitum), APP-PS1-ADF (APP-PS1 double transgenic mice underwent alternate day fasting). APP-PS1-ad libitum (double transgenic mice fed ad libitum), 3xAD-ADF (triple transgenic mice underwent alternate day fasting), and 3xAD-ad libitum (triple transgenic mice fed ad libitum).
